# Supplementary material for: BAP1 Loss Induces Senescence and Enhances the Response to Radiation Therapy and Senolytics
Source: bioRxiv. 2026 Feb 9:2026.02.05.704104. Preprint. [Version 1] doi: 10.64898/2026.02.05.704104 (PMC12918832; doi:10.64898/2026.02.05.704104)
Supplement: Supplement 2 [file NIHPP2026.02.05.704104v1-supplement-2.pdf]

bioRxiv preprint doi: https://doi.org/10.64898/2026.02.05.704104; this version posted February 9, 2026. The copyright holder for this preprint (which was not certified by peer review) is the author/funder, who has granted bioRxiv a license to display the preprint in perpetuity. It is made available under aCC-BY 4.0 International license.

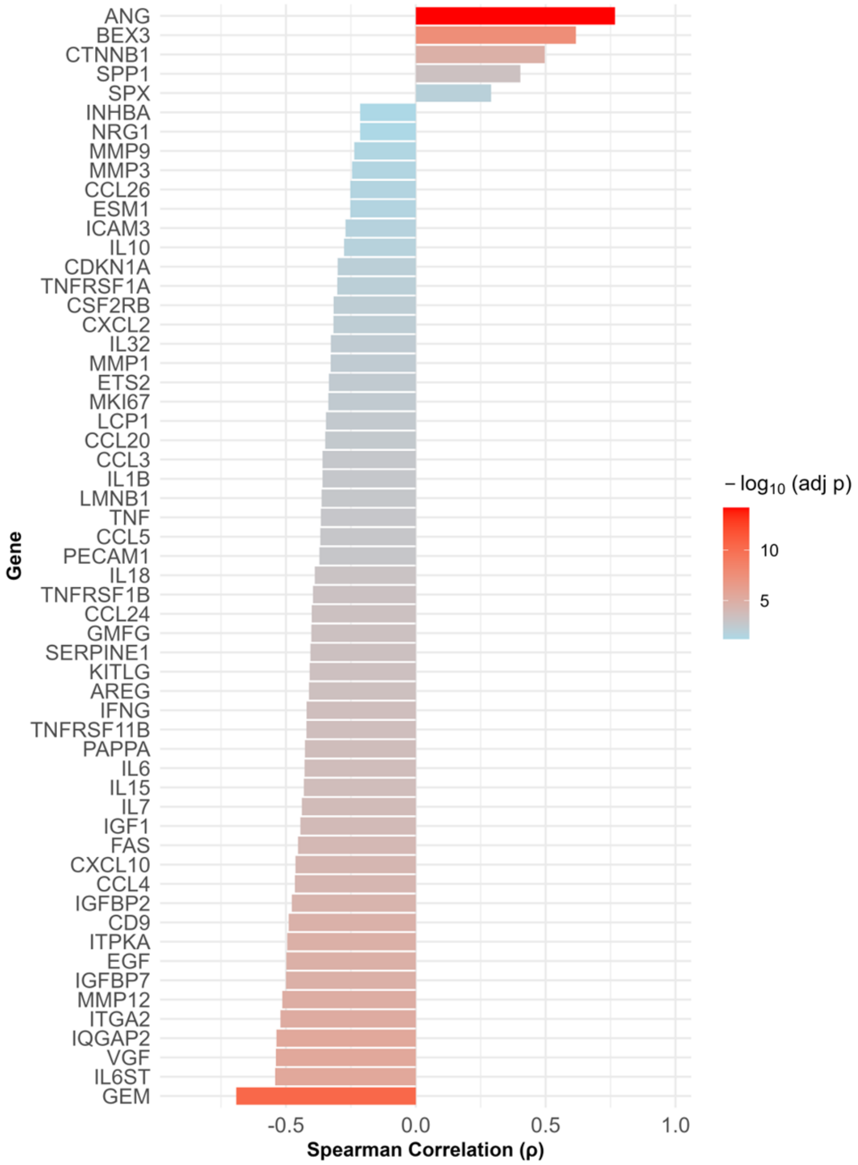

Supplementary Figure 1. BAP1 mRNA level is negatively correlated with the mRNA levels of a key senescence-related genes. Bar plot displaying Spearman correlation analysis between BAP1 mRNA and key senescence-associated genes mRNA levels in The Cancer Genome Atlas uveal melanoma dataset. The color intensity of each bar represents statistical significance ($-\log_{10}$ adjusted p value).

bioRxiv preprint doi: https://doi.org/10.64898/2026.02.05.704104; this version posted February 9, 2026. The copyright holder for this preprint (which was not certified by peer review) is the author/funder, who has granted bioRxiv a license to display the preprint in perpetuity. It is made available under aCC-BY 4.0 International license.

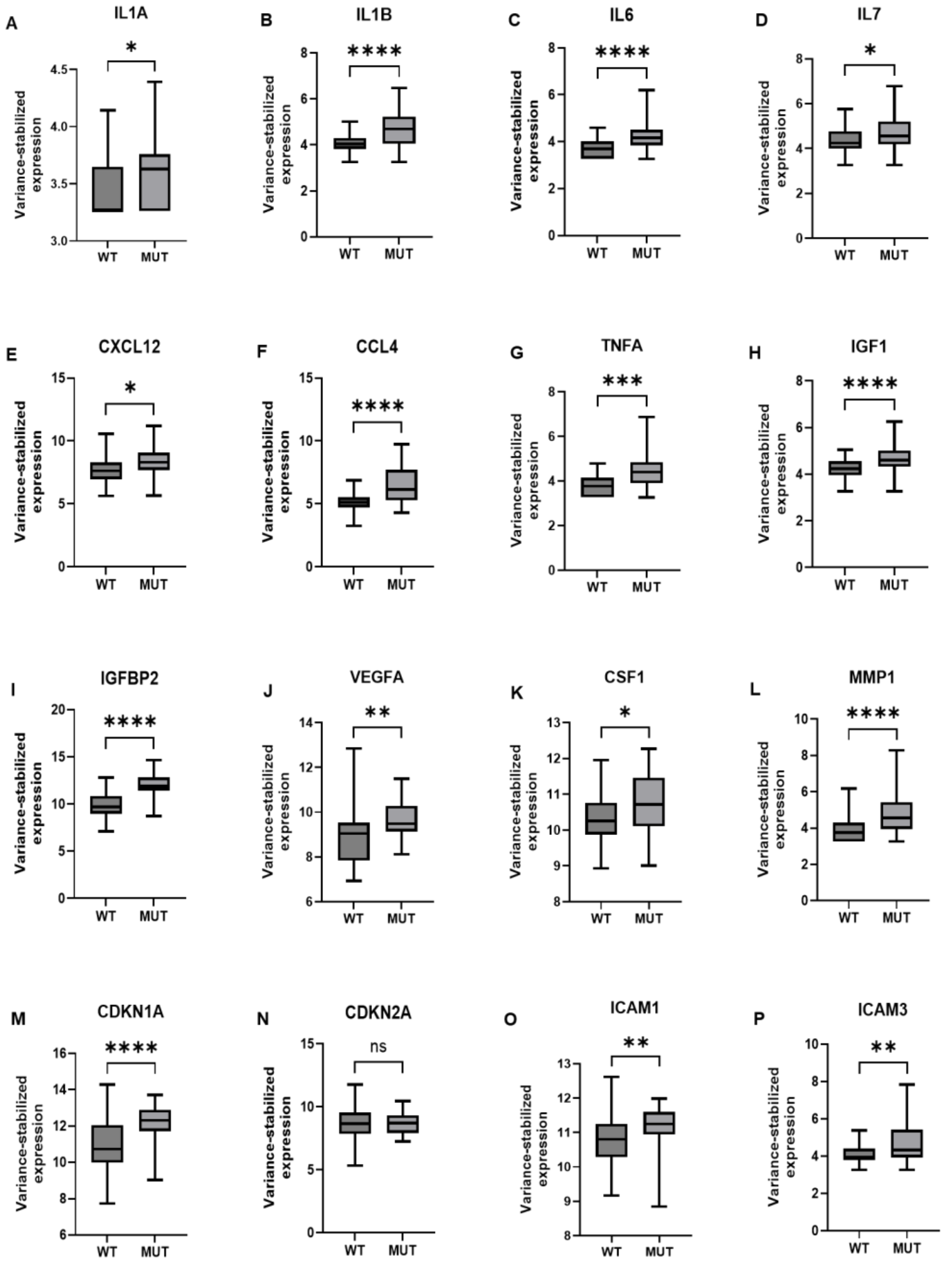

Supplementary Figure 2. BAP1 alterations are associated with upregulation of cytokines, chemokines, and growth factors in The Cancer Genome Atlas uveal melanoma dataset. (A – G) Boxplots showing mRNA expression levels of key cytokines and chemokines including IL1A (A), IL1B (B), IL6 (C), IL7 (D), CXCL12 (E), CCL4 (F), and TNFA (G). Boxplots depicting mRNA expression levels of key growth factors including IGF1 (H), IGFBP2 (I), VEGFA (J), and CSF1 (K). Boxplot analyses showing mRNA expression levels of additional key senescence markers including MMP1 (L), CDKN1A (M), CDKN2A (N), ICAM1 (O) and ICAM3 (P). Shapiro–Wilk test was used to determine the normal distribution of data. Student's t and Mann–Whitney U tests were used to calculate the statistical significance between parametric and nonparametric groups, respectively. Data are presented as means ± SD. (* $P \leq 0.05$; ** $P < 0.01$; *** $P < 0.001$; **** $P < 0.0001$). ns = nonsignificant.

bioRxiv preprint doi: https://doi.org/10.64898/2026.02.05.704104; this version posted February 9, 2026. The copyright holder for this preprint (which was not certified by peer review) is the author/funder, who has granted bioRxiv a license to display the preprint in perpetuity. It is made available under aCC-BY 4.0 International license.

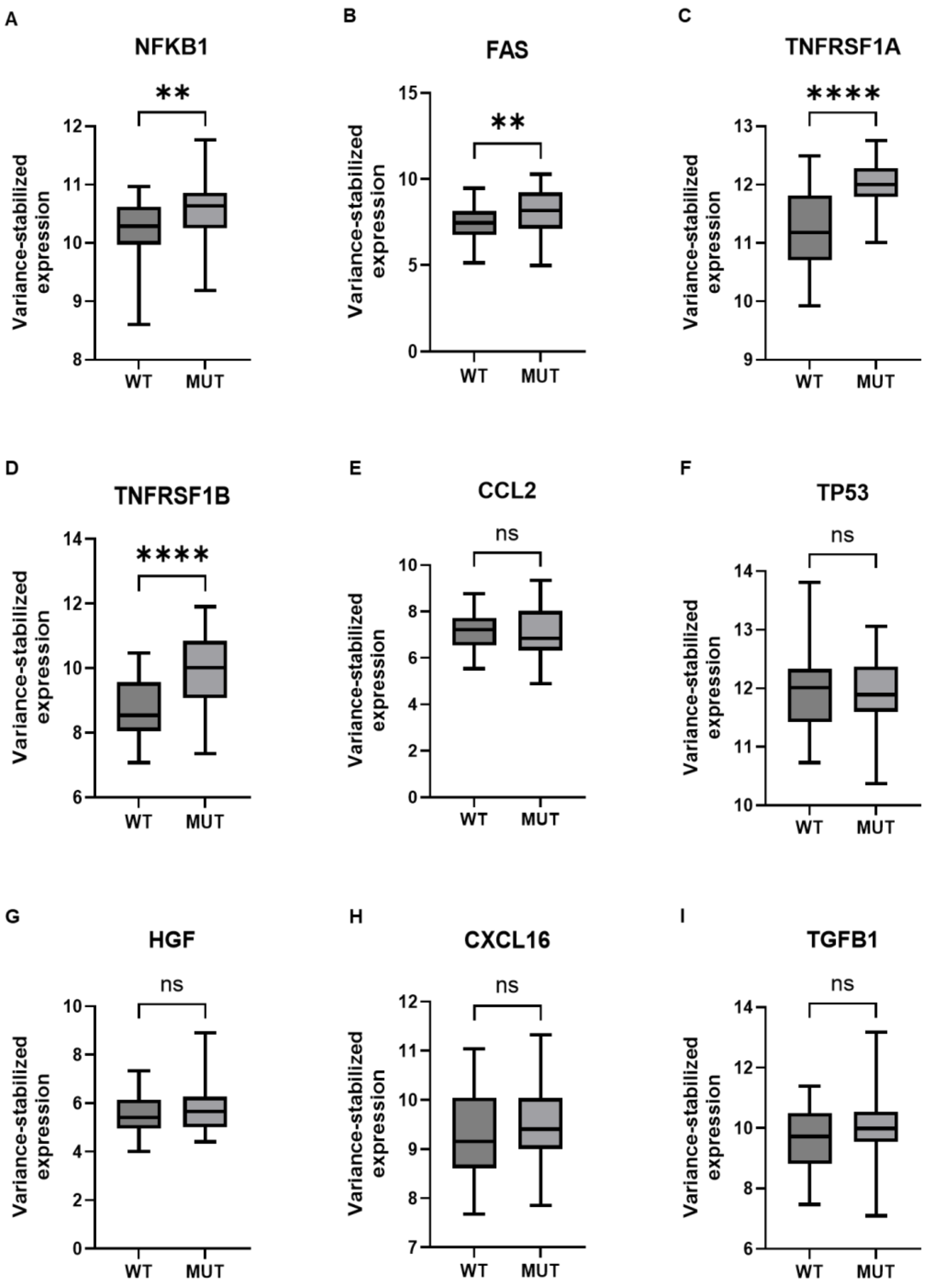

Supplementary Figure 3. BAP1 alterations are associated with upregulation of key inflammatory mediators in The Cancer Genome Atlas uveal melanoma dataset. (A – D) Boxplots showing mRNA expression levels of key inflammatory mediators including NFKB1 (A), FAS (B), TNFRSF1A (C), and TNFRSF1B (D). Boxplot analyses showing mRNA expression levels of additional key senescence markers including CCL2 (E), TP53 (F), HGF (G), CXCL16 (H) and TGFB1 (I). Shapiro–Wilk test was used to determine the normal distribution of data. Student's t and Mann–Whitney U tests were used to calculate the statistical significance between parametric and nonparametric groups, respectively. Data are presented as means ± SD. (** $P < 0.01$; **** $P < 0.0001$). ns = nonsignificant.

bioRxiv preprint doi: https://doi.org/10.64898/2026.02.05.704104; this version posted February 9, 2026. The copyright holder for this preprint (which was not certified by peer review) is the author/funder, who has granted bioRxiv a license to display the preprint in perpetuity. It is made available under aCC-BY 4.0 International license.

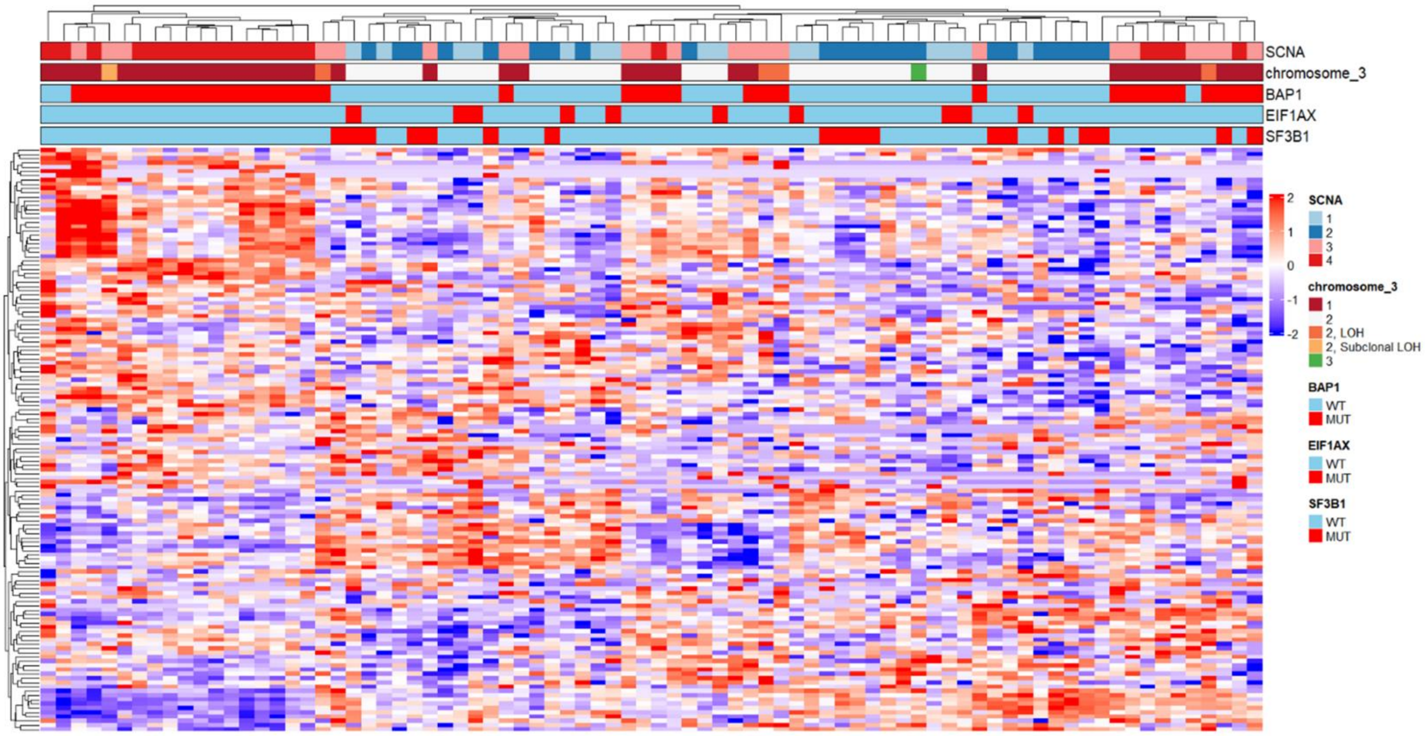

Supplementary Figure 4. Unsupervised clustering of the entire senescence-related genes (SenMayo + SenNet) in The Cancer Genome Atlas uveal melanoma cohort. Heatmap showing differential expression of key senescence-associated genes across UM samples (n = 80), with red and blue colors indicating up- and downregulated genes, respectively. Each column corresponds to an individual patient. Expression values were TMM normalized and z-score was scaled per gene. SCNA = somatic copy number variations. LOH = loss of heterozygosity. WT = wildtype. MUT = mutant.

bioRxiv preprint doi: https://doi.org/10.64898/2026.02.05.704104; this version posted February 9, 2026. The copyright holder for this preprint (which was not certified by peer review) is the author/funder, who has granted bioRxiv a license to display the preprint in perpetuity. It is made available under aCC-BY 4.0 International license.

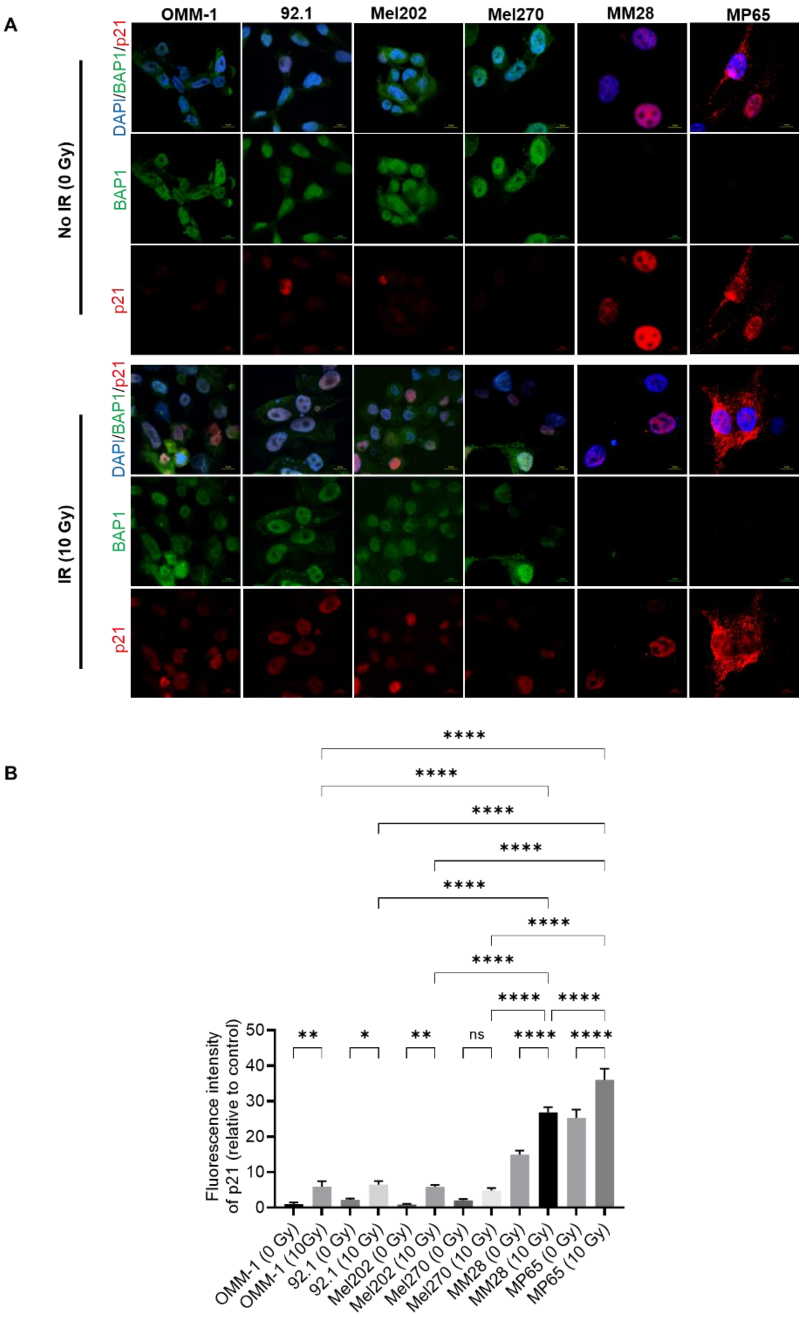

Supplementary Figure 5. The effect of ionizing radiation on senescence-related p21 protein expression in different BAP1 wild-type and mutant uveal melanoma cell lines. (A) Images representing immunofluorescence analysis of p21 in six uveal melanoma cell lines before (upper panel) and after (lower panel) exposure to ionizing radiation. Scale bar = 10 μm. (B) Quantitative image analysis of p21 fluorescence intensity. Data are presented as means (SD). Analysis of variance was used to determine the statistical significance of differences between groups. Multiple comparisons were performed using Tukey's honestly significant difference post hoc test. (* $P \leq 0.05$; *** $P < 0.001$; **** $P < 0.0001$). ns = nonsignificant. IR = ionizing radiation. Gy = gray.

bioRxiv preprint doi: https://doi.org/10.64898/2026.02.05.704104; this version posted February 9, 2026. The copyright holder for this preprint (which was not certified by peer review) is the author/funder, who has granted bioRxiv a license to display the preprint in perpetuity. It is made available under aCC-BY 4.0 International license.

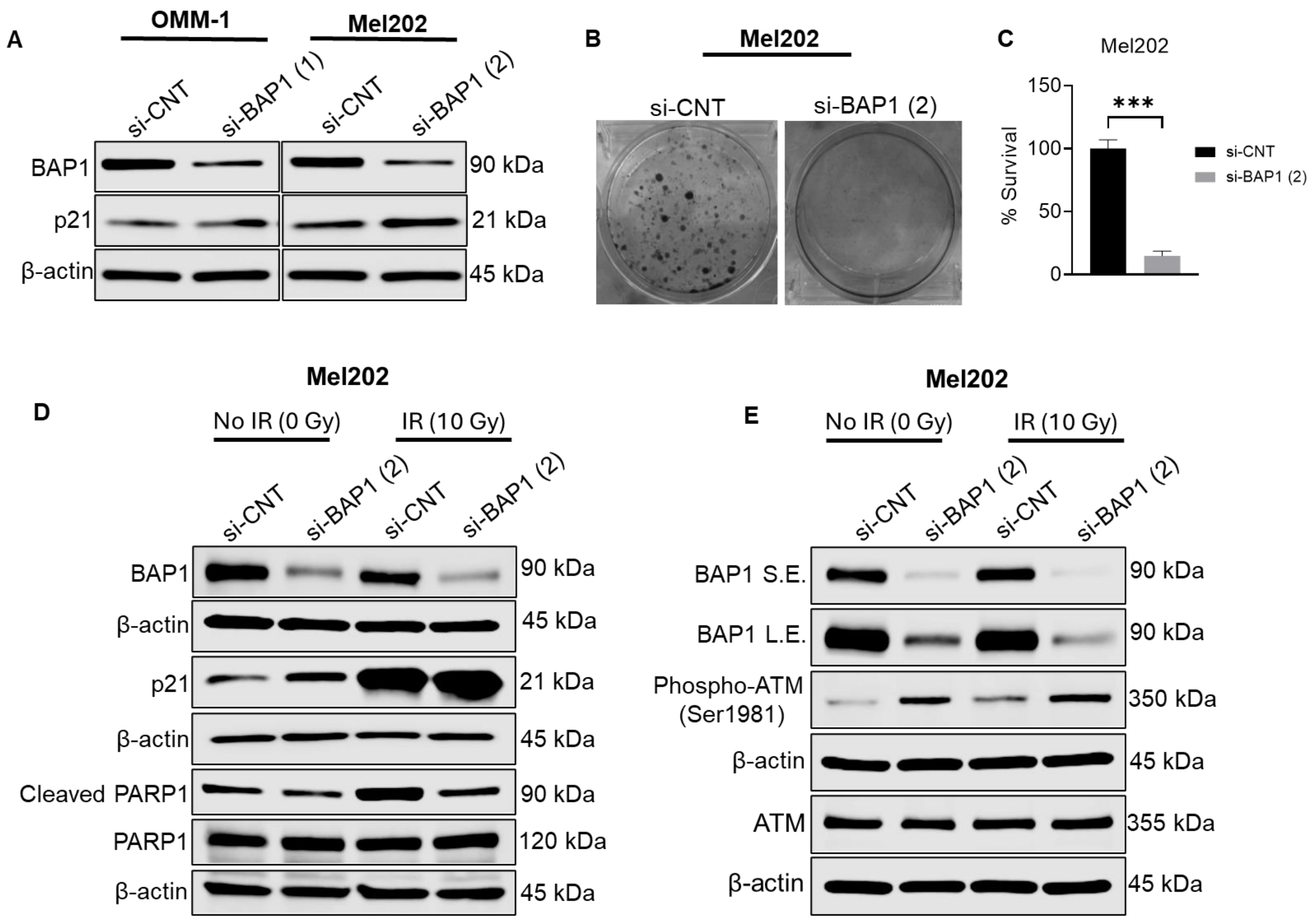

Supplementary Figure 6. BAP1 loss suppresses proliferation and enhances the protein expression of p21, phospho-ATM, and phospho-P53 in Mel202 cells. (A) Western blot showing the protein expression of BAP1 and p21 after transfecting OMM-1 and Mel202 cell lines with control or BAP1 siRNA. Beta actin was used as a loading control. (B) Images representing clonogenic assay after transfecting Mel202 cells with control or BAP1 siRNA (1). (C) Quantitative analysis of colony formation images in OMM-1. Data are presented as percent means (SD) (*** *P* < 0.001). Unpaired Student's t test was used to determine the significant difference between two groups. (D) Western blot analysis showing the protein expression of BAP1, PARP1, cleaved PARP, and p21 in Mel202 cell line. (E) Western blot analysis showing the protein expression of BAP1, phospho-ATM (Ser1981), and ATM in Mel202 cells transfected with control or BAP1 siRNA with or without exposure to ionizing radiation. β-actin was used as a loading control. si-BAP1 = BAP1 siRNA. IR = ionizing radiation. Gy = gray. si-CNT = control siRNA.
